# Treatment of Parkinson disease by acupuncture combined with medicine based on syndrome differentiation from the perspective of modern medicine: A review

**DOI:** 10.1097/MD.0000000000034278

**Published:** 2023-07-28

**Authors:** Xue Xia, Xu Dong, Kaiqing Li, Jing Song, Dan Tong, Yang Liu, Yixiao Han, Dongyan Wang

**Affiliations:** a Heilongjiang University of Traditional Chinese Medicine, Harbin, Heilongjiang Province, China; b Second Affiliated Hospital of Heilongjiang Traditional Chinese Medicine, Harbin, Heilongjiang Province, China.

**Keywords:** acupuncture treatment, biomarkers, combination of acupuncture and medicine, motor symptoms, non-motor symptoms, Parkinson disease, prevention and treatment with traditional Chinese medicine

## Abstract

As a multifactorial degenerative disease, Parkinson disease (PD) causes tremor, gait rigidity, and hypokinesia, which interfere with normal life. Because the disease is usually discovered in the late stage of complete degeneration of neurons, it can greatly delay treatment and even eventually lead to death. Therefore, the diagnosis of this disease is very challenging, and it is gratifying that substantial progress has been made in the development of optical coherence tomography (OCT) as a diagnostic biomarker for this disease, and genetic and imaging tests have become part of routine protocols in clinical practice. In the cognition of traditional Chinese medicine (TCM), this disease belongs to deficiency in origin and excess in superficiality, which is always caused by deficiency of liver and kidney, deficiency of qi and blood, and is closely related to wind, fire, phlegm and blood stasis. A large number of studies have shown that TCM can effectively treat motor and non-motor symptoms of PD, combat oxidative stress and reduce inflammatory response, and improve the quality of life of patients. Based on the pathophysiological mechanism of PD, this paper discusses the treatment of PD by TCM acupuncture combined with medicine based on syndrome differentiation.

## 1. Introduction

Parkinson disease (PD) is a common neurological disease and the second most common neurodegenerative disease worldwide, with more than 6 million patients worldwide.^[1]^ PD describes a syndrome characterized by stiffness, tremor, and bradykinesia, with motor and non-motor symptoms, the incidence of which increases steadily with age.^[2]^ The impairment of voluntary motor control leads to the signs and symptoms of dyskinesia. Bradykinesia, hypokinesia, postural instability, body stiffness, hunching, tremor at rest, often present with gait disturbance, stiffness of arms, legs and trunk, poor balance and coordination, bilateral vocal cord paralysis, at extreme and worsening levels. These motor characteristics were used to monitor the response to treatment and to assess the progression of PD.^[3]^

Pathological features of PD include neural inclusions in the form of Lewy bodies and Lewy nerve fibers, loss of cells in the substantia nigra (SN) and other brain regions. In view of aggregated and misfolded α-synuclein, Braak.^[4]^ A spreading pattern of Lewy pathology was proposed, starting from the caudal brainstem and progressing peripherally, but this spreading may not occur in all patients.

Age is the most important risk factor for PD, which affects about 0.3% of patients in industrialized countries. It is rarely seen in patients under 40 years of age, and its incidence increases with age. It is estimated that 3% of the population over the age of 80 may be affected by the disease.^[5]^ Men are more affected than women, and several studies have shown that the onset of PD is on average 2 years earlier in men than in women, with an incidence ratio of about 3:2.^[6]^ While living in rural areas and exposure to pesticides are risk factors, attracting tobacco and coffee seems to have a protective effect.^[6]^

There is no convincing evidence that early initiation of treatment has any effect on the progression of the disease, nor does any treatment confer neuroprotection. The decision to treat is based on the impact of symptoms.^[7]^ Medication is also aimed at improving symptoms. Based on the above limitations, the treatment of PD patients in modern medicine is not satisfactory, and complementary and alternative medicine treatment models and methods are needed to help treat the disease. Based on the pathophysiological mechanism of modern medicine, this paper discusses the treatment of PD with the combination of traditional Chinese medicine (TCM) and acupuncture.

## 2. Clinical diagnostic criteria

In order to improve the diagnostic accuracy of PD, the International and Movement Disorder Association has proposed a new set of criteria to replace the revised version of the most commonly used diagnostic criteria for Queen Square Brain Bank Criteria in the past few decades.^[8]^ These new criteria are based on expert neurological examination showing the presence of bradykinesia and at least 1 additional basic motor feature (rigidity or classic asymmetric 5 Hz resting tremor) in PD, plus the application of supportive and exclusionary features. In contrast to the list of motor features of the Queen Square Brain Bank Criteria, the Movement Disorders Association lists a number of non-exclusive clinical features that are uncommon in PD and should raise suspicion of potential alternative diagnoses (so-called “red flags”). Based on the presence of support and the absence of exclusion, as well as the presence or absence of a “red flag,” the Movement Disorders Association criteria classify the definitive diagnosis of PD into 2 levels, clinically established and clinically probable. The former establishes a set of criteria designed to maximize specificity while potentially sacrificing sensitivity, while the latter criteria are designed to improve sensitivity.^[9]^ Subject to space limitations, these criteria^[2,9,10]^ The specific content is not the focus of this article, so it is not written. For details, please refer to the relevant literature.

### 2.1. Non-motor symptoms of PD

The clinical hallmark of PD is the movement syndrome, which is characterized by bradykinesia, resting tremor, and stiffness, in addition to changes in posture and gait. Dyskinesia leads to progressive disability, daily life is affected, and quality of life is reduced. Although typical motor symptoms occur early and are the mainstay of diagnostic criteria, postural uncertainty and increasing gait difficulties, as well as the development of dysphagia and dysarthria, drive the development of dyskinesia. PD is considered a motor disorder, but almost all patients have a variety of non-motor symptoms, including hyposmia, constipation, voiding dysfunction, orthostatic hypotension, memory loss, depression, pain, and sleep disorders. Typical motor symptoms of PD are associated with nigral degeneration and striatal dopamine depletion, while non-motor symptoms may be associated with neurodegeneration of other structures, including non-motor symptoms are common in the early stages, although intense and disturbing for some patients, but observational studies have shown that these symptoms are mild in most cases.^[11]^ Its severity increases with the course of the disease.^[12]^ In particular, cognitive decline and hallucinations are common causes of admission and hospitalization in the late stages of the disease.

### 2.2. Prodromal phase of PD

Currently, the diagnosis of PD is based on clinical criteria requiring the presence of bradykinesia along with at least 1 motor symptom such as tremor, stiffness, or postural instability.^[13]^ The incubation period from the onset of the first non-motor symptoms of PD to meeting the current diagnostic criteria for PD can range from 5 to 20 years, which is called the prodromal period of PD.^[13]^ At this stage, patients already have neurodegenerative symptoms or signs of PD, but do not meet current diagnostic criteria. Therefore, this stage is often overlooked or misdiagnosed, but this period provides an opportunity for early diagnosis, pathophysiological research, and improved treatment of the disease, which may slow or prevent the occurrence of motor symptoms in PD.^[14,15]^ In recent years, the research on early PD has been deepened, including clinical motor and non-motor symptoms, fluid and tissue biomarkers, genetic susceptibility factors and neuroimaging imaging. This article will review the clinical manifestations, auxiliary examinations and biomarkers of PD in the prodromal stage, so as to deepen the understanding of PD, which will contribute to the early diagnosis and improved treatment of PD, so as to alleviate the burden of caregivers caused by PD in the late clinical stage.

Biomarkers of prodromal PD have been extensively studied and are considered to be high-risk markers for early diagnosis of PD. In recent years, more and more biomarkers have been proposed and confirmed, and various biomarkers have their unique predictive ability and lead time, which brings new hope for the identification and neuroprotective treatment of early PD. At present, most of the non-motor symptoms have been confirmed to be associated with a high risk of PD, and even closely related to different clinical phenotypes, but the field of imaging, genetic and other humoral markers still needs extensive and in-depth exploration.

### 2.3. Epigenetic-based diagnosis and treatment

PD is a progressive neurodegenerative disease. However, when many patients are diagnosed for the first time because of typical motor symptoms, about 50% of dopaminergic neurons have been irreversibly lost. Therefore, the discovery of biomarkers for the early diagnosis of PD is of great significance for treatment and prognosis. The highly similar methylation modification patterns in brain and peripheral blood suggest that peripheral blood may be an effective proxy for brain methylation levels.^[16]^ Previous studies have suggested that SNCA and LRRK2 hypomethylation in peripheral leukocytes can be used as potential biomarkers for early diagnosis of PD.^[16]^ In addition, miRNAs obtained from serum, plasma, and circulating blood cells have also been proposed as possible PD biomarkers.^[17]^ However, there are also some difficulties in the current study of PD epigenetic biomarkers. First, blood samples cannot fully represent the methylation level of brain tissue, and the methylation level of different brain regions may be different. There are a large number of dopaminergic neurons in the SN of PD patients, which may affect the results; Secondly, the use of levodopa can reverse the hypomethylation status of SNCA gene introns, so methylation studies of PD patients who have been treated with levodopa may underestimate the methylation level of PD patients themselves.^[17]^ Moreover, many current studies on methylation use bisulfite transformation sequencing or chip detection, which can not effectively identify 5mC and 5hmC, and may mistake 5hmC status for 5mC.

## 3. Biomarkers of PD

At present, one of the important limitations in the treatment of PD is that the disease can only be detected in the late stage. Despite rigorous efforts in patient management and clinical research, methods for diagnosis, refining prognosis, predicting individual response to therapeutic interventions, and tracking disease are suboptimal. Therefore, there is an urgent need for diagnostic biomarkers with high sensitivity and specificity to help diagnose diseases.^[18]^

Biomarkers can indicate a specific disease state of an organism, assess disease progression, and evaluate treatment efficacy. It can be a physical, chemical, or biological parameter.^[19]^ In the diagnosis of PD, although no single biomarker is recommended, they can be reasonably combined to predict the status and progression of the disease. To sum up, due to the difficulty of diagnosing PD at this stage, it is very difficult for the academic community to find biomarkers.

### 3.1. Imaging biomarkers

In the diagnosis of PD, more and more imaging biomarkers are used to support clinical observation. Imaging biomarkers include molecular imaging, transcranial ultrasonography, magnetic resonance imaging (MRI), and optical coherence tomography (OCT).

#### 3.1.1. Molecular imaging.

Molecular imaging technologies are not just those that provide anatomical images. In MS, molecular imaging is a type of medical imaging that provides a picture of events occurring in the body at the cellular and molecular level and measures chemical and biological processes compared to other imaging techniques. In PD, it measures the function of neurons and other brain tissue that use neurotransmitters such as dopamine. The molecular imaging procedure is noninvasive, safe, and painless. This involves an imaging device, an imaging agent, and a probe. There are 2 widely used imaging techniques focused on dopamine, Dopamine Transporter Single Photon Emission Computed Tomography and Fluorodopa Positron Emission Tomography.^[20]^

#### 3.1.2. Transcranial ultrasonography.

Transcranial ultrasonography is a noninvasive and painless technique that can be performed without anesthesia.^[21]^ Transcranial ultrasonography is helpful in visualizing the paranasal and diencephalic hyperechoic signals in PD, which represent the dysfunction of the dopaminergic SN pathway.^[22]^ Hyperechoic refers to the echo produced by fatty deposits during ultrasound examination of an organ. Hyperechoic SN in midbrain PD patients is thought to be associated with increased iron concentration, which leads to oxidative stress and further loss of dopaminergic neurons causing PD. Increased iron levels in the SN have been reported in PD patients, reflecting disturbances in the brain iron balance.^[23]^ Elevated SN iron is an invariable feature of PD and is considered a sufficient cause of neurodegeneration in PD.^[24]^

#### 3.1.3. Magnetic resonance imaging.

MRI of PD specifically demonstrates the presence of increased iron in the SN of PD patients. One of the most developed MRI markers is iron loading, using T2/T2^*^relaxation measurement.^[25]^ T2/T2^*^imaging of PD SN using T2/T2^*^imaging, changes in the relaxation time constant can be measured as a surrogate for increases in concentration in PD. These brain changes in PD were discovered with a powerful MRI (3 Tesla scanner), which produces twice the magnetic field strength of a 1.5 Tesla machine and 10 to 15 times the strength of an open MRI scanner.^[26]^ MRI has become a standard technique routinely performed in PD patients to rule out secondary causes and provide specific information to help diagnose neurodegenerative diseases such as PD. Studies have found that 30% of dopaminergic neurons in the SN of PD patients have died in the early stage. Neuromelanin is present in dopaminergic neurons in the SN, where it binds toxic metal ions, such as iron, and plays a protective role in cells. However, neuromelanin is obviously absent in the SN of PD patients, which provides some evidence for the imaging diagnosis of PD.

#### 3.1.4. OCT as a recent biomarker for PD.

OCT provides accurate cross-sectional imaging of the internal structure of biological tissues to reveal various internal retinal or optic neuropathies. Vision is one of the non-motor systems altered in PD. Neurochemical analysis of the eyes of PD patients showed a decrease in retinal dopamine concentration, resulting in decreased vision. In the human retina, dopamine is released by a group of amine cells in the inner layer of the retina, which communicate with other cells and play a role in transmitting visual information through the retina. Therefore, retinal nerve fiber thickness is a potential biomarker for the diagnosis of PD.^[27]^ Retinal assessment was performed by detecting apoptotic retinal cells and OCT.^[28]^

### 3.2. Biochemical biomarkers

Biochemical biomarkers can be studied in cerebrospinal fluid (CSF) or blood. CSF is an accessible source of brain-derived protein.^[29]^ It is separated by the blood-brain barrier, which provides nutrients to brain tissue and filters waste from the brain interstitium. In PD, the blood-brain barrier is disrupted, so CSF can be investigated as a potential biomarker.^[30]^ Additionally, CSF remains in intimate contact with the extracellular space of the brain. A study^[31]^ CSF is routinely collected in patients with neurologic symptoms and should closely reflect changes in the brain of PD patients. In this study, the researchers describe a scalable and sensitive mass spectrometry-based (MS) proteomics workflow for CSF proteome analysis. From 2 independent cohorts of more than 200 people, their workflow repeatedly quantified more than 1700 proteins from a minimal amount of CSF. Machine learning screened out changes in OMD, CD44, VGF, PRL, and MAN2B1 in PD patients or significantly correlated with clinical scores. They also found a signature of enhanced neuroinflammation in LRRK2 G2019S carriers, showing elevated levels of CTSS, PLD4, and HLA proteins. The comparison with the urinary protein group previously obtained by the investigators showed a large overlap of PD-related changes, including lysosomal proteins, which opened new avenues for better understanding of the pathogenesis of PD.

#### 3.2.1. Brain-derived neurotrophic factor (BDNF).

BDNF is a member of the neurotrophic factor family, which is widely and highly expressed in the brain, especially in the hypothalamus, limbic system and hippocampus,^[32]^ and is mainly produced by glutamatergic neurons and glial cells.^[32]^ In the periphery, BDNF is expressed by skeletal muscle cells, smooth muscle cells, endothelial cells, and activated immune cells.^[22]^ Platelets are the most important peripheral reservoir of BDNF, with BDNF levels 100 to 1000 times higher than in neurons.^[32]^ Studies have shown that the level of BDNF may be a risk factor for cognitive impairment in PD patients, and the level of serum BDNF can be used as a clinical indicator for predicting cognitive impairment in PD patients.^[32]^ Recent studies have shown that the level of serum BDNF is negatively correlated with the degree of depression in PD patients, and the decrease of BDNF level may be involved in the pathophysiological mechanism of PD depression.^[32]^ Gene regulation or physical exercise can increase the expression of BDNF, which can reduce muscle tension and cognitive deficits in PD patients, further suggesting that BDNF is a neuroprotective factor for PD.

#### 3.2.2. Neurofilament light chain protein (NfL).

It is still controversial whether NfL levels in the CSF or blood are higher in patients with PD than in healthy adults.^[33]^

However, clinical observations have shown that NfL levels are significantly higher in patients with atypical parkinsonism such as progressive supranuclear palsy, multiple system atrophy and corticobasal degeneration with neurodegeneration and significant involvement of myelin axons than in patients with PD.^[34]^ At present, blood NfL detection has been widely used in the study of various neurological diseases. Although the expression level of NfL has high accuracy in distinguishing PD from atypical parkinsonism, it is not yet able to identify the specific type of atypical parkinsonism.^[35]^ With the emergence of disease-modifying treatment strategies for PD, it is important to stratify patients at an early stage of the disease. Some researchers^[35]^ believe that the elevation of serum NfL level indicates that patients change from the preclinical stage of PD to the clinical stage; at the same time, NfL can be used to monitor the impact of drugs on the disease process.

#### 3.2.3. α-Synuclein and other lysosomal enzymes.

Lysosomal dysfunction and impairment is increasingly recognized as a central event in the pathophysiology of PD and thus as a potential biomarker of PD. Lysosomes are part of the cellular waste disposal system, and their dysfunction is involved in the pathogenesis of PD. Clearly, this autophagy-lysosome system is responsible for the hydrolysis of dysfunctional proteins, and in the case of damaged lysosomes, ASN aggregation was observed to be a common feature of PD. This ASN aggregation also involves post-translational modifications and certain unknown factors. Certain lysosomal enzymes, such as glucocerebrosidase and owl protease, have been reported in lysosomal dysfunction. Therefore, the detection of lysosomal enzymes and proteins in CSF can be used as a biomarker for PD. However, relying solely on the activity of CSF lysosomal enzymes cannot differentiate PD from other diseases. Combining CSF lysosomal markers with ASN species and indicators of mitochondrial dysfunction, inflammation, and other pathological proteins in PD may lead to a more accurate diagnosis.^[36]^

## 4. Cognition of PD in TCM

In the ancient medical books of TCM, there is no clear reference to PD. According to the classification, PD basically belongs to the category of “tremor disease,” “arrest disease” or “tremor arrest disease” in TCM.^[37]^ PD patients with resting tremor can be diagnosed as “Chan disease,” and those with muscle tension and bradykinesia can be diagnosed as “Ju disease,” both of which are obviously diagnosed as “Chan-Ju disease.” In some previous diagnostic guidelines, this disease has been diagnosed as “tremor disease” in TCM. However, because a considerable number of patients have never shown limb symptoms and head tremor in the course of the development of the disease, this diagnosis does not seem to be in line with the actual situation and needs to be revised.

In the Ming Dynasty, Sun Yikui wrote Chishui Xuanzhu.^[38]^ It is believed that the pathogenesis of this disease in TCM is “wood, fire and earth are abundant, kidney yin is not full” Excess is phlegm-fire, and deficiency is kidney deficiency. Later, with the deepening of research, Chinese medicine practitioners of all Dynasties have developed the pathogenesis theory of this disease in combination with their own clinical practice. At present, TCM has formed a certain consensus on the pathogenesis of this disease, that is, this disease belongs to deficiency in origin and excess in superficiality, and deficiency of liver and kidney and deficiency of qi and blood are the root of the disease. It is true that wind, fire, phlegm and blood stasis are the symptoms.^[37]^ If the disease occurs for a long time, it will be transformed into a mixture of deficiency and excess.

## 5. TCM treatment of PD

### 5.1. Study on TCM prescription for PD

As we all know, Chinese medicine prescription treatment of this disease follows the principle of syndrome differentiation and treatment, according to the consensus of experts, the disease is divided into the following syndromes: Deficiency of yin and blood and loss of nourishment of tendons^[39]^; Deficiency of yin and blood and internal movement of liver wind^[40]^; Type of stagnation of Shaoyang qi and internal disturbance of phlegm-fire^[41]^; Deficiency of middle-jiao qi and internal movement of liver wind^[42]^; Deficiency of both yin and Yang.^[42]^ To a certain extent, these classification criteria can be used as a reference to guide the clinical practice of TCM.

Xu Zijin^[43]^ to study the clinical effect of Zhichan Decoction (Radix Astragali, Radix Salviae Miltiorrhizae, Ramulus Uncariae Cimicifugae, Rhizoma Anemarrhenae, Radix Paeoniae Alba, Radix et Rhizoma Rhei Preparata and Rhizoma Cimicifugae) on liver and kidney deficiency PD and its influence on intestinal flora, 72 patients with liver and kidney deficiency PD were enrolled in the study, 36 patients in the treatment group and 36 patients in the control group. After 12 weeks of treatment, Zhichan Decoction could improve the clinical symptoms of PD, increase the abundance of Hemophilus, and decrease the abundance of Ackermann and Porphyromonas.

Song Lei^[44]^ from January 2021 to January 2022, 60 patients with early PD in Handan Hospital of Traditional Chinese Medicine were randomly divided into control group and observation group with 30 cases in each group. To observe the effect of Pingchan Shujin Decoction (Radix Astragali, Radix Angelicae Sinensis, Radix Achyranthis Bidentatae, Radix Ginseng, Rhizoma Polygonati, Radix Rehmanniae Preparata, Rhizoma Acori Tatarinowii, Radix Polygalae, Radix Paeoniae Alba, Carapax et Plastrum Testudinis, Carapax Trionycis, Concha Ostreae, Cortex Cinnamomi and Cornu Cervi Pantotrichum) combined with western medicine on dopamine, neurotrophic factors and. It is found that Pingchan Shujin Decoction helps to improve the level of dopamine in the brain, protect neurons and improve cognitive function by up-regulating the level of neurotrophic factors and down-regulating the level of inflammatory factors.

Han Xuejuan^[45]^ based on the PI3K/AKT/mTOR signaling pathway, the efficacy of Yangxin Dingji Capsule (Hebei Yongfeng Pharmaceutical Co., Ltd., specification: 0. 5g/capsule, product batch number: 20170902) in the treatment of PD with depression was investigated. 102 PD patients with depression were randomly divided into control group and study group, with 51 cases in each group. The control group was treated with conventional western medicine, while the study group was treated with Yangxin Dingji Capsule on the basis of the control group. It was finally found that Yangxin Dingji Capsule had a good effect on PD patients with depression, which could alleviate the development of the disease, improve non-motor symptoms, and reduce depression, possibly through affecting the PI3K/AKT/mTOR signaling pathway.

### 5.2. Study on acupuncture and moxibustion for PD

The study found that the mechanism of acupuncture treatment of PD is mainly reflected in the following aspects.^[46]^ Inhibition of dopaminergic neuron apoptosis; Inhibit the aggregation of α-prominent nuclear protein; Inhibition of oxidative stress; Control nerve inflammation.

Ma Xue^[47]^ to observe the effect of early electroacupuncture intervention on ionized calcium adapter protein-1, tyrosine hydroxylase and tumor necrosis factor-α in PD mice, and to explore the mechanism of electroacupuncture in the treatment of PD from the perspective of neuroinflammatory response. Nine-week-old male C57BL/6J mice were randomly divided into blank group, model group and electroacupuncture group, with 8 mice in each group. Studies have found that early electroacupuncture intervention delayed the time of rotenone-induced dyskinesia in PD mice, reduced the loss of dopaminergic neurons in the SN, and played a good neuroprotective role in the degenerative changes of PD, which may be related to the improvement of intestinal inflammatory response by acupuncture, inhibition of microglial activation, and reduction of neuroinflammatory response.

Guo Jie^[48]^ to observe the effect of electroacupuncture (EA) on the expressions of synaptophysin, postsynaptic density protein 95, Ca2 + binding adaptor molecule 1^+^, CD68^+^microglia and complement related protein in the hippocampal CA1 region of a mouse model of PD and dementia (PDD). To explore the partial mechanism of “Xiusanzhen” in improving the memory impairment of PDD. Male C57BL/6 mice were randomly divided into blank group, sham operation group, model group and electroacupuncture group, with 10 mice in each group. Finally, it was found that “Xiusanzhen” could alleviate the learning and memory impairment of PDD model mice and improve the synaptic plasticity of hippocampal CA1 region, which may be related to its reduction of the expression of C1q and C3 in hippocampal CA1 region and the phagocytosis of microglia.

Liu Yanyang^[49]^ to observe the EA on the apoptosis of dopaminergic neurons in rats with PD and to explore its possible molecular mechanism. SD rats were randomly divided into sham operation group, model group, electroacupuncture group, MCC950 group and electroacupuncture + MCC950 group with 12 rats in each group. The ameliorative effect of EA on dopaminergic neurons in PD rats may be related to the inhibition of neuronal apoptosis mediated by nucleotide-binding oligomerization domain-like receptor protein 3/Caspase-1.

About moxibustion, Zhang Yumin^[50]^ to explore the effect of Linggui Bafa moxibustion on the clinical rehabilitation treatment of PD, and to provide clinical basis for moxibustion assisted rehabilitation treatment of PD. In this study, 30 patients with PD were randomly divided into treatment group (15 cases) and control group (15 cases), and both groups received routine drug treatment and nursing in the Department of Neurology, and symptomatic management and treatment were carried out according to the patients’ complications. The control group was treated with exercise therapy (posture training, balance training, gait training), hand function training, activities of daily living training and psychotherapy, while the treatment group was treated with acupoint moxibustion therapy of Linggui Bafa on the basis of the control group. It was finally found that the total effective rate of the treatment group was significantly better than that of the control group. After 4 weeks of treatment, the Unified Parkinson disease rating scale (UPDRS) score and total score of the treatment group were improved better than those of the control group, and the HY grade of the former was lower than that of the latter (*P* < .05). Conclusion Linggui Bafa acupoint moxibustion is effective, safe and reliable in the adjuvant treatment of PD.

### 5.3. Study on treatment of PD with acupuncture combined with medicine

In the long-term medical practice of TCM, the academic community has gradually explored the treatment mode of acupuncture combined with medicine for PD. Compared with the single treatment mode, the combination of acupuncture and medicine not only absorbs the characteristics of quick effect, simplicity and convenience of acupuncture treatment, but also embodies the advantages of lasting curative effect and seeking the root of the disease of TCM, which is the main treatment method and mode for PD in the field of TCM.

Liu Xuejiao^[51]^ to observe the effect of Sangzhen Yangshen Formula (Fructus Mori, Fructus Ligustri Lucidi, Radix Angelicae Sinensis, Periostracum Cicadae, Radix Polygoni Multiflori Preparata, Radix Paeoniae Alba, Fructus Tribuli, Bombyx Batryticatus, Carapax et Plastrum Testudinis, Ramulus Uncariae Cum Uncis, Radix Salviae Miltiorrhizae, and Radix Glycyrrhizae Tablets) combined with acupuncture (Baihui, Wangu, Fengfu, Fengchi. 76 cases of PD with deficiency of liver-yin and kidney-yin syndrome were randomly divided into treatment group and control group, 38 cases in each group. The study found that Sangzhen Yangshen Formula plus acupuncture combined with levodopa and benserazide tablets could improve the gait balance and walking ability of PD with deficiency of liver-yin and kidney-yin syndrome, and improve the quality of life, which may be related to alleviating nerve inflammatory reaction and promoting nerve repair.

Song Xi^[52]^ to analyze the curative effect of Bushen Zhichan Decoction combined with acupuncture in the treatment of sleep disorders in PD and its impact on blood glutamate and cystatin C levels. Methods one hundred twenty-four PD patients with liver and kidney yin deficiency complicated with sleep disorders were randomly divided into observation group and control group, and the control group received conventional western medicine treatment. The observation group was treated with Bushen Zhichan Fang (Fructus Lycii, Radix Paeoniae Alba, Ramulus Uncariae cum Uncis, Herba Cistanches, Radix Rehmanniae, Carapax et Plastrum Testudinis, Radix Codonopsis, Fructus Cannabis, Radix Ophiopogonis, Carapax Trionycis, Rhizoma Gastrodiae, Colla Cornus cervi, Colla Corii Asini, Rhizoma Polygonati, Fructus Corni, and Radix Glycyrrhizae Preparata) and acupuncture (After treatment, the sleep quality [PD sleep scale, Pittsburgh sleep quality index], quality of life [PD quality of life scale 39], clinical efficacy of TCM, plasma glutamate and serum cystatin C levels before and after treatment, daily average dosage of madopar and adverse reactions were observed. It was finally found that on the basis of conventional western medicine treatment combined with Bushen Zhichan Fang and acupuncture can effectively improve the clinical symptoms of PD patients with sleep disorders, improve their quality of life, and the treatment is safe and worthy of clinical promotion.

Cai Guolin^[53]^ to explore the clinical efficacy of mind-regulating acupuncture (bilateral Xinshu, Ganshu, Pishu, Hegu, Taichong, Baihui, Shenting, Shenmen, Neiguan) combined with Bushen Huoxue Shugan Decoction (Radix Puerariae, Radix Cyathulae, Radix Salviae Miltiorrhizae, Scorpio, Fructus Corni, Herba Ecliptae, Radix Astragali, Fructus Ligustri Lucidi, Rhizoma Gastrodiae, Radix Bupleuri, Radix Curcumae, Rhizoma Chuanxiong. 108 patients with PD and depression admitted to the hospital from March 2019 to March 2021 were selected and divided into the observation group and the control group according to the random number table. The patients in the control group were treated with dobethazine tablets combined with pramipexole, and the patients in the observation group were additionally treated with mind-regulating acupuncture combined with Bushen Huoxue Shugan Decoction on the basis of the treatment in the control group.Both groups were treated for 3 months. The scores of UPDRS, Hamilton Depression Scale, TCM symptoms, clinical efficacy, serum levels of human cartilage glycoprotein 39 (YKL-40) and BDNF and adverse reactions were compared between the 2 groups. Finally, it was found that the acupuncture method of regulating mind combined with Bushen Huoxue Shugan Decoction could improve the clinical symptoms of PD patients with depression, and the curative effect was exact, at the same time, it could reduce the inflammatory reaction and promote the recovery of cognitive function, which was safe and reliable.

Wang Xiaodi^[54]^ to observe the clinical efficacy of Dihuang Ziyin Bushen Formula (Radix Rehmanniae Preparata, Cortex Eucommiae, Fructus Corni, Carapax et Plastrum Testudinis, Radix Paeoniae Alba, Radix Notoginseng, Bombyx Batryticatus, and Radix Glycyrrhizae) combined with acupuncture (Radix Baihui, Sishencong, Fengchi, Hegu, Yanglingquan, Taichong, Shenshu, Xuehai, and Sanyinjiao) in the treatment of PD with. Methods Eighty patients with PD of liver and kidney deficiency syndrome were randomly divided into observation group (n = 42) and control group (n = 38). The control group was treated with conventional therapy, while the observation group was treated with Dihuang Ziyin Bushen Decoction combined with acupuncture on the basis of the control group. UPDRS scores, syndrome scores, PD quality of life scale 39 scores of patients with PD, and the levels of superoxide dismutase, malondialdehyde and interleukin-1β were compared between the 2 groups before and after treatment. To study the effect of Dihuang Ziyin Bushen Prescription combined with acupuncture on PD patients with liver and kidney deficiency syndrome, which can not only effectively alleviate the symptoms of liver and kidney deficiency, but also resist oxidative stress and reduce inflammatory reaction, and is conducive to improving the quality of life of patients (Table 1).

**Table 1:** Research methods, methods and results of traditional Chinese medicine treatment of PD.

Experimental researchers	Group	Treatment	Conclusion
Xu Zijin	Treatment group	Stopping tremor soup	After 12 wk of treatment, Zhichan Decoction could improve the clinical symptoms of PD, increase the abundance of Haemophilus, and decrease the abundance of Ackermann and Porphyromonas.
	Control group	Antitremor placebo	
Song Lei	Treatment group	Pingchan Shujin Decoction	Pingchan Shujin Decoction helps to increase the level of dopamine in the brain, protect neurons and improve cognitive function by up-regulating the level of neurotrophic factors and down-regulating the level of inflammatory factors.
	Control group	Carbidopa and levodopa controlled-release tablets	
Han Xuejuan	Treatment group	Yangxin Dingji Capsule	Yangxin Dingji capsule has a good effect on PD patients with depression, which can alleviate the development of the disease, improve non-motor symptoms, and reduce depression, which may play a role by affecting the PI3K/AKT/mTOR signaling pathway.
	Control group	Levodopa and Benserazide Tablets	
Ma Xue	Blank group	No treatment	Early electroacupuncture intervention delayed the onset of rotenone-induced dyskinesia in PD mice, reduced the loss of dopaminergic neurons in the substantia nigra, and played a good neuroprotective effect on the degenerative changes of PD, which may be related to the improvement of intestinal inflammatory response, inhibition of microglial activation, and reduction of neuritis.
	Model Group	Subcutaneous injection of rotenone	
	Electroacupuncture group	Shenting, Tianshu, Quchi, Shangjuxu	
Guo Jie	Blank group	No treatment	“Xiusanzhen” can alleviate the learning and memory impairment of PDD model mice and improve the synaptic plasticity of hippocampal CA1 region, which may be related to its reduction of the expression of C1q and C3 in hippocampal CA1 region and the phagocytosis of microglia.
	Sham operation group	Fake surgery	
	Model Group	Subcutaneous apomorphine	
	Electroacupuncture group	Smell 3 needles	
Liu Yanyang	Model Group	6-OHda 2-point injection method	The ameliorative effect of EA on dopaminergic neurons in PD rats may be related to the inhibition of neuronal apoptosis mediated by NLRP3/Caspase-1.
	Sham operation group	Fake surgery	
	Electroacupuncture group	Electroacupuncture at Taichong and Fengfu	
	MCC950 group	Intraperitoneal injection MCC950	
	Electroacupuncture + MCC950 group	Intraperitoneal injection on the basis of electroacupuncture MCC950	
Zhang Yumin	Treatment group	The control group was treated with acupoint moxibustion therapy based on Linggui Eight Methods.	After 4 wk of treatment, the improvement of UPDRS scores and total scores in the treatment group was better than that in the control group, and the HY grade in the treatment group was lower than that in the control group (*P* < .05). Conclusion Linggui Bafa acupoint moxibustion is effective and safe in the adjuvant treatment of Parkinson disease.
	Control group	Exercise therapy, hand function training, activities of daily living training and psychotherapy	
Liu Xuejiao	Treatment group	Sang Zhen Yang Shen Fang combined with acupuncture	Sangzhen Yangshen Recipe combined with acupuncture and levodopa and benserazide tablets can improve the gait balance, walking ability and quality of life in patients with liver and kidney yin deficiency syndrome of Parkinson disease, which may be related to the reduction of nerve inflammatory reaction and the promotion of nerve repair.
	Control group	Levodopa and Benserazide Tablets	
Song Xi	Treatment group	Kidney-tonifying and Tremor-Stopping Decoction plus Acupuncture and Benserazide	On the basis of conventional western medicine treatment combined with Bushen Zhichan Fang and acupuncture can effectively improve the clinical symptoms of PD patients with sleep disorders, improve their quality of life, and the treatment is safe, worthy of clinical promotion.
	Control group	Levobarazide	
Cai Guolin	Treatment group	Tiaoshen needling combined with Bushen Huoxue Shugan Decoction	Mind-regulating acupuncture combined with Bushen Huoxue Shugan Decoction can improve the clinical symptoms of patients with Parkinson disease complicated with depression, and the curative effect is exact, at the same time, it can reduce the inflammatory reaction and promote the recovery of cognitive function, which is safe and reliable.
	Control group	Tobaserzide	
Wang Xiaodi	Treatment group	Combined application of kidney-tonifying and trembling-stopping prescription and acupuncture	Dihuang Ziyin Bushen Decoction combined with acupuncture can not only effectively alleviate the symptoms of liver and kidney deficiency, but also resist oxidative stress and reduce inflammatory reaction, which is conducive to improving the quality of life of patients.

NLRP3 = nucleotide-binding oligomerization domain-like receptor protein 3, PD = Parkinson disease, PDD = Parkinson disease and dementia, UPDRS = Unified Parkinson disease rating scale.

## 6. Conclusion

TCM has a long history and rich medical experience in the treatment of PD. However, the clinical intervention trials of TCM have not yet provided clear evidence for the treatment of PD. Cell models and animal models of PD constructed in vivo and in vitro cannot perfectly reflect the clinical stage of research, or lack of further clinical validation. In addition, most importantly, the design of TCM in PD clinical trials often fails to meet the conditions of randomized double-blind, which is in urgent need of change.

It is worth noting that Wang Xuelin^[55]^ based on the theory of 5 movements and 6 qi, the law of birth luck of PD patients was analyzed, and the etiology and pathogenesis of PD were discussed from the perspective of 5 movements and 6 qi. They collected the information of 13,878 PD patients, and selected 100,000 non-PD patients as controls to compare the prevalence of PD under different luck, and used the theory of luck to study. The results showed that PD patients were greatly influenced by the seasonal factors of Yunqi, and the pathogenesis may be related to cold coagulation and blood stasis, weakness of the spleen and stomach, deficiency of kidney qi and stagnation of liver qi.

Acupuncture is effective in the treatment of PD, especially in the combination of acupuncture and medication. However, there are still some limitations in the current clinical studies. First of all, the subjectivity of the evaluation of some effect indexes may reduce the credibility of acupuncture efficacy, and objective evaluation methods are worth exploring. Secondly, few studies have increased or decreased the appropriate acupoints according to different pathological stages or symptoms, and compared the differences in efficacy. Also, acupuncture protocols and stimulation parameters vary from trial to trial. Future studies on acupuncture treatment of PD should be carried out with higher methodological quality.

The combination of acupuncture and medicine, because the TCM prescription itself is difficult to achieve complete objectification and standardization, coupled with the increasing difficulty of combined therapy in clinical research, to achieve objective, clear and clear research results, is a test of the ability of researchers.

To sum up, acupuncture and TCM are both effective strategies for the treatment of PD, and the combination of acupuncture and TCM can improve the efficacy and quality of life of patients, but the existing studies can not rule out the possibility of this treatment as a placebo effect, which needs to be verified by higher treatment studies.

## Author contributions

**Conceptualization:** Xue Xia.

**Data curation:** Xue Xia, Xu Dong, Kqiqing Li, Jing Song, Dan Tong, Yang Liu, Yixiao Han, Dongyan Wang.

**Formal analysis:** Xue Xia.

**Funding acquisition:** Xue Xia.

**Investigation:** Xue Xia.

**Methodology:** Xue Xia.

**Project administration:** Xue Xia.

**Resources:** Xue Xia.

**Software:** Xue Xia.

**Supervision:** Xue Xia.

**Validation:** Xue Xia.

**Visualization:** Xue Xia.

**Writing – original draft:** Xue Xia.

**Writing – review & editing:** Xue Xia.
